# PGC-1α-Targeted Therapeutic Approaches to Enhance Muscle Recovery in Aging

**DOI:** 10.3390/ijerph17228650

**Published:** 2020-11-21

**Authors:** Jonathan J. Petrocelli, Micah J. Drummond

**Affiliations:** Department of Physical Therapy and Athletic Training, University of Utah, 520 Wakara Way, Salt Lake City, UT 84108, USA; jonathan.petrocelli@utah.edu

**Keywords:** disuse, sarcopenia, metformin, leucine, resveratrol, skeletal muscle, PGC-1alpha

## Abstract

Impaired muscle recovery (size and strength) following a disuse period commonly occurs in older adults. Many of these individuals are not able to adequately exercise due to pain and logistic barriers. Thus, nutritional and pharmacological therapeutics, that are translatable, are needed to promote muscle recovery following disuse in older individuals. Peroxisome proliferator-activated receptor gamma coactivator 1-alpha (PGC-1α) may be a suitable therapeutic target due to pleiotropic regulation of skeletal muscle. This review focuses on nutritional and pharmacological interventions that target PGC-1α and related Sirtuin 1 (SIRT1) and 5′ AMP-activated protein kinase (AMPKα) signaling in muscle and thus may be rapidly translated to prevent muscle disuse atrophy and promote recovery. In this review, we present several therapeutics that target PGC-1α in skeletal muscle such as leucine, β-hydroxy-β-methylbuyrate (HMB), arginine, resveratrol, metformin and combination therapies that may have future application to conditions of disuse and recovery in humans.

## 1. Introduction

Skeletal muscle disuse in older individuals (>65 years old) increases fall risk, hospitalization, and chronic disease development and accelerates age-induced muscle loss (sarcopenia) [1,2]. During recovery from disuse (e.g., surgery, injury, illness), older individuals experience delayed muscle size and strength recovery compared to their younger counterparts [3,4,5,6,7,8], a phenotype recapitulated in aged rodents [9,10]. Disuse-induced muscle atrophy and weakness and prolonged recovery from disuse in aging contribute to reduced life- and healthspan as skeletal muscle is important for whole-body glucose regulation [11], postural stability/balance to prevent falls [12], and strength to perform activities of daily living [13]. Thus, there is a need to prevent the consequences of muscle disuse and enhance recovery in older adults. This need was reported in 2006 [14], yet minimal change has occurred in clinical practice. Exercise is currently the only employed clinical remedy to prevent (prehab) or enhance recovery from disuse. Exercise is undoubtably beneficial. However, exercise may not be practical in those who cannot partake due to pain or logistic barriers [15,16]. Therefore, there is a need for alternate, yet translational therapeutic approaches to prevent muscle atrophy and enhance recovery with aging. Repurposing pharma- and nutraceuticals is an attractive solution given the time to translatability. Peroxisome proliferator-activated receptor gamma coactivator 1-alpha (PGC-1α) and the related Sirtuin 1-5′AMP-activated protein kinase (SIRT1–AMPKα) signaling axis may be a promising therapeutic signaling mechanism to target as it has pleiotropic effects alleviating related muscle aging and disuse complications [17,18,19,20], which may potentially enhance muscle functional recovery. This review will briefly cover the role of PGC-1α in regulating muscle size and function during disuse, and thoroughly review translatable therapies that promote SIRT1–AMPKα–PGC-1α activity that may potentially prevent the consequences of age-related disuse while bolstering muscle recovery.

## 2. Role of PGC-1α in Skeletal Muscle Aging, Atrophy and Recovery from Disuse

**Figure 1:**
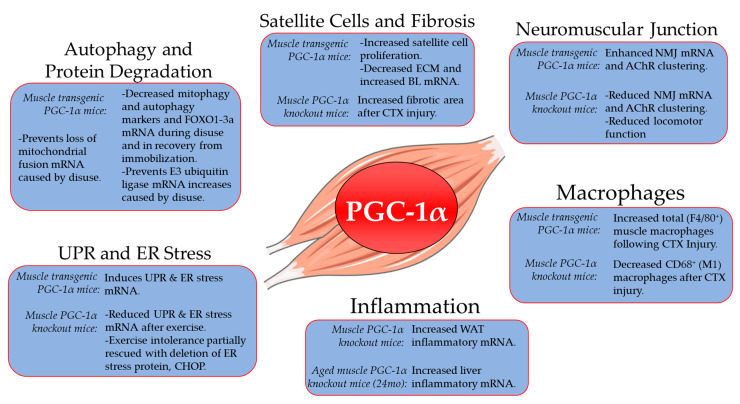
PGC-1α roles in skeletal muscle tissue beyond mitochondrial biogenesis. Evidence is presented from skeletal muscle-specific PGC-1α gain (transgenic) and loss (knockout) of function studies. Information for autophagy and protein degradation derived from [24,25,26,27]; satellite cells and fibrosis [20,29]; neuromuscular junction [18,28]; macrophages [20]; inflammation [30]; UPR and ER stress [19]. PGC-1α, peroxisome proliferator-activated receptor gamma coactivator 1-alpha; FOXO1, forkhead box protein O1; FOXO3a, forkhead box protein 3a; mRNA, messenger RNA; ECM, extracellular matrix; BL, basal lamina; CTX, cardiotoxin; NMJ, neuromuscular junction; AChR, acetylcholine receptor; WAT, white adipose tissue, UPR; Unfolded protein response, ER; Endoplasmic Reticulum, CHOP; C/EBP homologous protein.

In skeletal muscle, PGC-1α is known for its regulation of mitochondrial biogenesis and function [21] and commonly regulated by SIRT1 and AMPKα [22,23]. Beyond regulating mitochondria function in muscle, PGC-1α plays a more vast role in muscle function such as regulating protein degradation and autophagy [24,25,26,27], neuromuscular junctions [18,28], endoplasmic reticular stress [19], satellite cell function [29], fibrosis [20], macrophage/inflammatory responses and necrosis [20,30] (Figure 1). Interestingly, these skeletal muscle facets are dysregulated features in aging and following disuse and recovery in humans and rodents [6,10,31,32,33,34]. Moreover, PGC-1α expression has been observed to be lower with advanced age [35,36,37,38,39], during atrophy-inducing conditions [24], during disuse [40,41,42,43], and in recovery from disuse [44,45]. Interestingly, PGC-1α overexpression in rodent muscle can prevent disuse-induced atrophy [24,46,47]. Similarly, muscle-specific PGC-1α ablation diminishes strength recovery following disuse [48]. A mechanism by which PGC-1α prevents disuse-induced atrophy is by reducing forkhead box O3a (FOXO3a) transcription [24] and subsequent expression of E3 ubiquitin ligases F-box protein 32 (MAFbx/atrogin-1) and muscle ring-finger protein 1 (MuRF-1/TRIM63). Muscle-specific PGC-1α overexpression also counteracts mitochondrial fusion dysfunction during disuse preventing E3 ubiquitin and autophagosome degradation pathways [26,27]. Therefore, PGC-1α in muscle appears to be a key player regulating muscle atrophy and recovery of muscle function by targeting proteolytic pathways.

Below we will present evidence that the SIRT1–AMPKα–PGC-1α signaling axis is targeted by several nutritional and pharmaceutical interventions and thus could be suitable translatable solutions to prevent disuse-induced muscle atrophy and improve recovery from disuse in older adults. Specifically, interventions that are readily translatable, have established safety and efficacy, and have demonstrated promise to prevent disuse and/or improve muscle recovery were reviewed.

## 3. Potential Nutritional Therapies

### 3.1. Leucine

The branch-chained amino acid (BCAA) leucine is well known to activate mechanistic target of rapamycin complex 1 (mTORC1) and promote muscle protein synthesis [49,50]. Leucine has also been shown to stimulate the SIRT1–AMPKα–PGC-1α signaling axis in skeletal muscle cells [51,52,53,54,55]. Though leucine treatment is not fully effective against muscle atrophy during disuse in rodents and humans [56,57,58], the role of leucine to enhance muscle recovery is a far less studied context. Indeed, leucine was able to accelerate muscle mass recovery after 8 days of limb immobilization in adult rats when combined with antioxidants/polyphenols [59] but the contribution of leucine nor the mechanism is not clear. Leucine increased skeletal muscle PGC-1α and messenger RNA (mRNA) related to mitochondrial biogenesis 3 h post oral gavage in rats, and in C2C12 myotubes 1 h of leucine treatment increased PGC-1α mRNA expression [53]. In C2C12 myotubes, incubation with the mTORC1 inhibitor rapamycin prevented the increase in PGC-1α expression caused by leucine treatment [53], suggesting dependency on mTORC1. On the other hand, skeletal muscle-specific PGC-1α KO mice reduced the phosphorylation of the downstream mTORC1 effector, eukaryotic translation initiation factor 4E-binding protein 1 (4EBP1) [60]. These results suggest that in skeletal muscle, leucine-induced PGC-1α expression depends on mTORC1 but a feedback loop involving mTORC1 and PGC-1α may also exist. Interestingly, piglet primary muscle cells treated with leucine for 3 days increased PGC-1α protein and mRNA (and promoted slow myosin heavy chain phenotype) yet was dependent on SIRT1 and AMPKα [61]. Similarly, C2C12 myotubes treated with leucine for 24 or 48 h also required SIRT1 and AMPKα to increase PGC-1α mRNA [55]. Together, these experiments suggest that there is an interconnected signaling network encompassing mTORC1, SIRT1, AMPKα, and PGC-1α in skeletal muscle with leucine treatment.

The dose and length of leucine administration (chronic vs. acute) appear to be critical for optimal PGC-1α activation. As mentioned above, acute leucine ingestion promotes PGC-1α expression, yet it is important to point out that a saturable effect of leucine occurred with a 24 h, 100 to 500 μM treatment on PGC-1α protein and oxygen consumption rate in C2C12 myotubes [62]. Thus, a limited PGC-1α activation may be achieved with a single dose of leucine but this has not been determined in skeletal muscle in vivo. In growing pigs, a diet doubling leucine ingestion beyond growing pig nutritional needs for 45 days resulted in BCAA imbalance (decreased isoleucine and valine), and reduced mRNA expression of genes related to BCAA metabolism. This chronic leucine treatment had no effect on PGC-1α mRNA expression. Rather this leucine diet induced a fast-twitch fiber phenotype through decreased oxidative type IIa and I myofibers and reduced growing piglet body mass and soleus muscle mass [63]. A similar chronic, augmented leucine diet in growing pigs also did not alter muscle PGC-1α protein yet promoted glycolysis and reduced fatty acid oxidation and oxidative phosphorylation [64]. Alternately, a 28 day, low-dose leucine diet given to mice with Lewis Lung Carcinoma increased PGC-1α protein, but muscle gastrocnemius and rectus femoris muscle size remained reduced in these mice [65]. In summary, acute leucine administration or lower leucine doses with chronic treatment may be optimal to promote skeletal muscle PGC-1α expression.

Overall, the vast breadth of muscle cell experiments clearly establishes leucine-induced PGC-1α induction. However, the lack of studies observing PGC-1α during muscle disuse and recovery in aging following leucine administration makes it difficult to interpret therapeutic potential. Current evidence suggests that leucine does not prevent disuse atrophy, putting into question whether PGC-1α expression is increased in these scenarios. Future research examining PGC-1α expression in response to leucine treatment in disuse and recovery is required to begin analyzing necessary dosing and length of leucine treatment required to promote PGC-1α and potentially rescue disuse-induced atrophy and enhance recovery in aging.

### 3.2. β-hydroxy-β-methylbuyrate (HMB)

β-hydroxy-β-methylbuyrate (HMB) is an active metabolite derived from leucine catabolism that may have promise to not only promote PGC-1α expression but also prevent disuse atrophy and enhance recovery in aging muscle. Since HMB is a product of leucine catabolism, it is not surprising that HMB effects are similar to leucine in stimulating protein synthesis through mTORC1 [66]. However, contrary to leucine, HMB may prevent amino acid (AA) imbalances. For instance, chronic HMB supplementation in growing piglet diets did not result in BCAA imbalance or glycolytic fiber shifts, but rather increased soleus muscle mass, whereas leucine in this study had the opposite affect (above) [63]. In young men 2.5 h after ingesting equal amounts of HMB or leucine, muscle mTORC1 signaling was activated with both treatments, although differently indicating potential diversity in mechanism of action [67]. Regarding disuse and recovery, older adults receiving HMB supplementation during 10 days of bed rest and 8 weeks of progressive resistance training had increased muscle mitochondrial complex proteins and triglyceride species during recovery compared to the control group [68]. Following 10 days of bed rest in older adults, HMB prevented a decrease in PGC-1α mRNA, and improved transcriptional profiles related to fibrosis, ribosomes, mitochondrial function and increased the mitochondrial membrane lipid species, cardiolipin [43]. In aged rats, HMB supplementation improved force production, muscle mass and myofiber cross-sectional area (CSA), and satellite cell proliferation during recovery from disuse and this was independent of changes in mTORC1 signaling [69]. Similarly, in aged rats recovering from disuse, HMB improved force production, CSA, and reduced skeletal muscle apoptosis and apoptotic signaling [70].

Together, HMB is capable of activating PGC-1α expression in skeletal muscle and prevents disuse atrophy and assists muscle recovery following disuse. However, it is unknown whether HMB activates PGC-1α directly or whether other nutrient sensors such as SIRT1, AMPKα, or mTORC1 play a role in HMBs ability to stimulate PGC-1α. Mechanistic studies are needed to determine whether PGC-1α is necessary for HMB effects. Such work will provide the foundation for determining whether HMB treatment can be modified or enhanced to promote PGC-1α expression and thus prevent disuse atrophy and promote recovery in aging.

### 3.3. Arginine

Individual essential amino acids (EAAs) such as arginine may also stimulate muscle PGC-1α and positively impact muscle health. Arginine administration for 42 days in mice or 3 days in C2C12 myotubes induces slow fiber transitions through increased PGC-1α, enhanced oxidative phosphorylation, and reduced glycolytic activity [71]. Interestingly, these effects were dependent on both SIRT1 and AMPKα [71]. Arginine supplementation in young adult rats during 8 weeks of exercise training resulted in enhanced PGC-1α expression, electron transport chain proteins, exercise performance and reactive oxygen species (ROS)-buffering enzymes, superoxide dismutase Cu-Zn (SOD1) and superoxide dismutase Mn (SOD2) [72]. These effects were exercise dependent, indicating that arginine in conjunction with muscle contractions (exercise) promotes an increase in PGC-1α expression [72]. Further, arginine supplementation during 14 days of hindlimb unloading in young rats prevented myofiber atrophy, increased soleus nitric oxide content and mTORC1 signaling, and reduced expression of MAFbx/atrogin-1 and MuRF-1 mRNA [73]. Additional studies are needed to improve our understanding of the mechanisms of arginine supplementation during muscle disuse and recovery to further interpret its therapeutic potential.

### 3.4. Resveratrol

Resveratrol (3,5,4′-trihydroxystilbene) is a polyphenolic phytoalexin produced by plant species in response to infection, stress, injury, bacteria, and UV irradiation [74,75]. Resveratrol is found in grape skin, seeds, and peanuts and can be produced by 70 different plant species [74,75]. Isolated resveratrol as a treatment results in a wide range of biological responses important to healthy aging including anti-glycation, anti-oxidant, anti-inflammatory, neuroprotective, and anti-cancer properties (see review [76]). In context of this review, resveratrol stimulates SIRT1–AMPKα–PGC-1α signaling, and thus may be an effective tool to use to offset disuse atrophy and improve muscle recovery in aging.

In aged rats, resveratrol treatment improved muscle mass and myofiber CSA during 14 days of recovery following 14 days hindlimb unloading [77]. In young female mice, following 7 days of unilateral limb immobilization, resveratrol prevented the loss in muscle mass, myofiber CSA and strength while concomitantly increased satellite cell content during 7 day recovery [78]. In another study in which young adult rats were given resveratrol 4 weeks prior to and during 14 days hindlimb unloading was effective to maintain body and muscle mass, strength, and prevent a glycolytic fiber shift and a decrease in SIRT1 and PGC-1α protein expression [79]. However, a study by Jackson et al. (2010) showed that, although resveratrol treatment given 1 week prior to and during 14 days hindlimb unloading ameliorated muscle mass and force loss, this was only effective in aged, but not in young adult rats [80]. An age-specific resveratrol response in this study versus what others have found in the young [79] is not completely clear but may be due to dosing and treatment duration (12.5 mg/kg/d for 3 weeks [80] compared to 400 mg/kg/d for 6 weeks [79]). Resveratrol treatment was also shown to reduce fibrosis and increase mRNA and protein expression of markers associated with extracellular matrix remodeling during 7 and 14 days of recovery in young rodents following gastrocnemius contusion injury [81,82]. The diverse muscle tissue analyzed (gastrocnemius, soleus, or plantaris), route of administration (oral gavage, intraperitoneal injection, or in food), dosing and timing of resveratrol may contribute to differences in effectiveness observed between young versus old rodents and between disuse and recovery studies.

The effectiveness of resveratrol on muscle function during disuse and recovery may be related to reduced fibrosis, increased ROS scavenging, preserved lipid substrate utilization and mitochondrial function, and augmented satellite cell abundance [76,77,78,79,80,81,82]. A common result of resveratrol treatment, irrespective of dose, administration route, and timing, is a muscle oxidative fiber type shift due to an upregulation of SIRT1 and PGC-1α expression [77,78,79,83,84,85]. The dependency of SIRT1 is noted by a reduced ability to prevent dexamethasone-induced L6 myotube atrophy in the presence of resveratrol when SIRT1 is blocked [86]. Resveratrol also appears to depend on forkhead box protein O1 (FOXO1) since resveratrol loses its potency in maintaining myotube size when FOXO1 expression is reduced during tumor necrosis factor-alpha (TNF-α) exposure in C2C12 cells [87]. Moreover, in C2C12 muscle cells, resveratrol increased PGC-1α expression through adiponectin receptor 1 (AdipoR1) regulation of calcium and SIRT1–AMPKα signaling [85], suggesting that, at least in muscle cell culture, resveratrol operates through an AdipoR1–SIRT1–AMPKα–PGC-1α signaling mechanism to prevent atrophy.

## 4. Nutritional Therapies Combined with Metformin

### 4.1. Metformin

Metformin is a first-line defense therapy for individuals with type 2 diabetes mellitus (T2D). Metformin is an inexpensive, well-tolerated, and widely prescribed drug which led to re-purposing appeal for metformin use in other diseases beyond T2D [88]. Mechanistically, metformin action is complex and may be dose and tissue dependent, but has been observed to signal through AMPKα and PGC-1α in many cell types, including hepatocytes and skeletal muscle [89,90,91,92,93,94,95,96]. The pleiotropic effects of metformin make it difficult to delineate an exact mechanism but contribute to excitement as a therapy for targeting multiple facets dysregulated with disease and aging [88]. A similar line of thinking in lieu of the evidence lend to metformin being a useful therapeutic option to promote recovery from disuse in older adults.

Currently, research exploring metformin to prevent disuse atrophy and promote muscle recovery in aging at least in rodents appears to be strong. During 7 days hindlimb immobilization in young adult rats, metformin treatment ameliorated muscle atrophy and prevented tissue fibrosis [97]. In another study, rats treated with metformin during 16 weeks of high fat diet was effective to counter myofiber atrophy, fibrosis and increased E3 ubiquitin ligases expression compared to the non-metformin treated group [98]. During recovery from burn injury, metformin protected against myofiber atrophy and muscle fat infiltration while increasing satellite cell abundance [95]. In another study, 21 days of metformin prior to cardiotoxin injury prevented muscle damage without altering embryonic myosin heavy chain or central nuclei content [99]. A total of 60 days of metformin treatment in mice improved aerobic capacity while 3 days of metformin exposure in C2C12 muscle cells promoted differentiation, anabolic signaling, and SOD2 protein expression [100]. Moreover, metformin treatment prevented mouse satellite cell exhaustion in vitro and in single myofibers [101], ROS emission in obese rats [102], human T cell inflammation in vitro [103], and enhanced muscle membrane stability through AMPKα in dysferlin deficient mice [104]. Together, these studies strongly suggest that metformin treatment may be suitable to prevent muscle damage or promote recovery through influencing multiple phenotypes and signaling pathways that are commonly altered with disuse.

Metformin is known to increase PGC-1α in skeletal muscle tissue and cells [91,94], but AMPKα dependance is unknown. In C2C12 myotubes, metformin is able to increase PGC-1α mRNA [94]. In mouse muscle, metformin increased AMPKα and PGC-1α expression in slow- and fast-twitch fibers, indicating that metformin can increase PGC-1α regardless of muscle fiber type [91]. Studies in hepatocytes support that metformin works through AMPKα to promote PGC-1α expression. In hepatocytes derived from liver-specific AMPKα1/2 null mice, the normal increase in PGC-1α with metformin are blunted [105]. Furthermore, blocking AMPKα with compound C prevented the metformin-induced increase in PGC-1α expression in hepatocytes [106]. These studies indicate that metformin can increase PGC-1α in skeletal muscle and may require AMPKα for this action but many mechanistic metformin studies in skeletal muscle are lacking.

When considering metformin therapy as a target of PGC-1α, one must consider the cell type, species (rodent vs. human), and dosing. For instance, in primary mouse hepatocytes treated with dexamethasone, 8 h of suprapharmacological metformin doses (1 and 2 mM) given with cyclic AMP decreased PGC-1α mRNA expression [105], whereas human primary hepatocytes exposed to 1 mM metformin for 48 h increased PGC-1α mRNA [106]. Further, in C2C12 myotubes, exposure of a single suprapharmacological metformin dose (2 mM) for 4, 8, 12 or 24 h increased PGC-1α mRNA expression, where a pharmacological dose (30 μM) did not during the same time course [94]. Metformin dosing also appears to alter mitochondrial function. Initially it was thought that metformin inhibits mitochondrial complex I to ameliorate enhanced glucose production in individuals with T2D [107]. More recent reports support that mitochondrial inhibition is caused by high metformin concentrations (≥1 mM) whereas clinically prescribed (50–80 μM) doses likely work through mechanisms independent of mitochondrial inhibition [92,93] and may actually improve mitochondrial function dependent on AMPKα [92,93]. However, therapeutic, prescribed doses of metformin (1.5–2 g/d) given to older adults blunted exercise-induced improvements in aerobic capacity through impaired mitochondrial respiration [108] and impaired resistance training muscle adaptations [109], suggesting that metformin may interfere with exercise training. Overall, given that metformin at higher doses may be consequential to mitochondrial function, it may be more beneficial to investigate lower metformin doses to prevent disuse atrophy and promote muscle recovery in aging.

### 4.2. Metformin Combination Therapies

The use of compounds discussed above (leucine, HMB, or resveratrol) combined with metformin or in combination with vitamins (vitamin D) has appeal to not only enhance treatment outcomes by achieving a synergistic effect, but also to lower metformin dosing and decrease the likelihood of metformin-induced side effects (primarily gastrointestinal distress). Metformin combined therapies have not been studied in the context of muscle disuse atrophy or recovery in aging. However, outside the focus of this review, some metformin combination therapies have been shown to improve muscle insulin sensitivity and alleviate metabolic dysfunction. Importantly, metformin combined therapies reveal SIRT1–AMPKα–PGC-1α signaling as a common target mechanism, suggesting that a combined nutraceutical-pharmaceutical therapy approach may be worth investigating to prevent disuse-induced muscle atrophy and promote recovery in aging.

For instance, metformin combined with vitamin D for 8 weeks in rats with hyperglycemia (2 week HFD + 1 streptozotocin (STZ) injection) resulted in increased muscle PGC-1α mRNA expression as well as decreased E3 ubiquitin ligases, fibrosis and sarcolemma abnormalities compared to metformin or vitamin D alone [110]. In insulin stimulated C2C12 cells, metformin combined with leucine for 2 or 24 h enhanced SIRT1 and AMPKα activity whereas metformin and leucine monotherapy did not. Moreover, the influence of metformin and leucine combination on AMPKα was blocked by SIRT1 pharmacological or siRNA-induced inhibition [111]. Metformin and leucine given during 6 weeks of HFD in mice enhanced glucose tolerance compared to a higher dose metformin monotherapy [112]. Metformin and resveratrol combination therapy increased AKT activation in triceps muscle, and this treatment resulted in improved glucose tolerance 4 weeks after a 9 week HFD intervention in mice [113]. Lastly, metformin combined with HMB and resveratrol resulted in increased oxygen consumption rate and AMPKα phosphorylation in C2C12 cells [114] which similarly also occurred with metformin-leucine combined therapy [111].

While understudied in skeletal muscle and during disuse and recovery in aging, metformin combination therapies may be promising SIRT1–AMPKα–PGC-1α signaling agonists to improve muscle function. Indeed, this field of research is in its infancy and other metformin combinations (such as with the compound sildenafil [115,116,117]) may prove to be interesting translational therapeutic targets.

## 5. Conclusions

**Figure 2:**
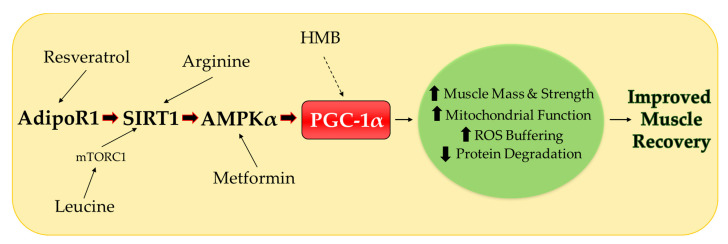
Proposed role of translational approaches on skeletal muscle SIRT1–AMPKα–PGC-1α signaling in preventing muscle disuse atrophy and promoting recovery in aging. Leucine, through mTORC1, activates SIRT1, which is required for increased AMPKα and PGC-1α. Arginine requires SIRT1 and AMPKα to promote PGC-1α. Metformin is dependent on AMPKα to increase PGC-1α in hepatocytes. However, this is unknown in skeletal muscle. Resveratrol works through AdipoR1 to increase SIRT1, AMPKα, and PGC-1α. HMB mechanistic studies on SIRT1–AMPKα–PGC-1α signaling in skeletal muscle are currently lacking but evidence suggests that HMB works through PGC-1α. PGC-1α, peroxisome proliferator-activated receptor gamma coactivator 1-alpha; AMPK, 5′ adenosine monophosphate-activated protein kinase; SIRT1, Sirtuin 1; AdpoR1, adiponectin receptor 1; mTORC1, mechanistic target of rapamycin complex 1; HMB, β-hydroxy-β-methylbuyrate; ROS, reactive oxygen species.

Preventing muscle disuse atrophy and enhancing muscle recovery in aging and utilizing translatable therapies is clinically important. PGC-1α is an intriguing therapeutic target as it regulates various aspects common to mechanisms associated with disuse atrophy and recovery. Therapies established for safety and efficacy such as leucine, HMB, arginine, resveratrol, metformin and combinations that promote SIRT1–AMPKα–PGC-1α signaling (Figure 2) are readily translatable options that may encourage enhanced muscle size and function during disuse and recovery periods with aging. A summary of the translational approaches model organism, tissue or cell type, dosage, route of administration and length of treatment on muscle PGC-1α can be found in Table 1.

## Figures and Tables

**Table 1:** Summary table of potential therapeutic approaches to influence muscle PGC-1α.

	Model	Age	Cell Type/Tissue	Additional Intervention	Dosage and Route of Administration	Length of Treatment	Influence on PGC-1α	Reference
**Leucine**	Mouse	—	C2C12 myotubes	—	2 mM in medium	1 d	↑ mRNA	51
	Mouse	—	C2C12 myotubes	—	1 and 2 mM in medium	1 d	↑ mRNA	52
	Rat	5 wks	Soleus EDL	—	135 mg/100 g BW via oral gavage	1 h and 3 h	↑ mRNA	53
	Mouse	—	C2C12 myotubes	—	20 mM in medium	1 h	↑ mRNA	53
	Mouse	—	C2C12 myotubes	—	2 mM in medium	1 d	↑ mRNA ↑ protein	54
	Mouse	—	C2C12 myotubes	—	0.5 mM in medium	2 d	↑ mRNA	55
	Pig	—	Primary myotubes	—	2 mM in medium	3 d	↑ protein	61
	Mouse	—	C2C12 myotubes	—	0.5 mM in medium	1 d	↑ protein	62
	Pig	11.4 wks (80 d)	Longissimus Dorsi Soleus	—	1.25% of diet	45 d	↔ mRNA	63
	Pig	7 wks	Longissimus Dorsi	—	1.66% and 2.1% of diet	14 d	↔ protein	64
	Mouse	9–10 wks	Gastrocnemius	Lewis Lung Carcinoma (LLC) injection	5% *w*/*w* supplemented in diet	28 d	↔ protein (control) ↑ protein (LLC group)	65
**HMB**	Pig	11.4 wks (80 d)	Longissimus Dorsi Soleus	—	0.62% of diet	45 d	↔ mRNA	63
	Human	66–67 yrs	Vastus Lateralis	10 d bed rest	3 g/d oral supplementation	15 d	↑ protein	43
**Arginine**	Mouse	3 wks	Tibialis Anterior	—	0.25, 0.5 and 1% supplemented in diet	42 d	↑ mRNA ↑ protein	71
	Mouse	—	C2C12 myotubes	—	0.5 mM in medium	3 d	↑ mRNA	71
	Rat	9–10 wks	Gastrocnemius	8 wk progressive treadmill running	62.5 mg/mL/d via oral gavage	56 d	↔ protein (control) ↑ protein (exercise group)	72
**Resveratrol**	Rat	32 mo	Plantaris	14 d hindlimb unloading and 14 d reloading	125 mg/kg/d via oral gavage and 0.05% supplemented in diet	35 d	↑ protein during unloading and reloading	77
	Rat	8 wks	Soleus	14 d hindlimb unloading	400 mg/kg/d via oral gavage	42 d	↔ mRNA ↑ protein (unloaded group)	79
	Rat	4–5 wks	Soleus Gastrocnemius	—	4 g/kg of diet	56 d	↔ protein	84
	Mouse	—	Triceps	HFD with resveratrol	4 g/kg of diet	56 d	↔ protein	84
	Mouse	—	C2C12 myotubes	—	20 μM in medium	6 h/d for 3 d	↑ protein	84
	Mouse	—	C2C12 myotubes	—	1, 5, and 10 μM in medium	1 d	↔ protein	84
	Mouse	15 wks	Extensor Digitorum Longus Soleus	—	400 mg/kg/d via oral gavage	84 d	↑ mRNA ↑ protein	85
	Mouse	—	C2C12 myotubes	—	20 μM in medium	—	↑ mRNA ↑ protein	85
**Metformin**	Rat	10 wks	Soleus Red gastrocnemius White gastrocnemius	—	1% of diet	14 d	↑ protein	91
	Mouse	—	C2C12 myotubes	—	2 mM in medium	4, 8, 12 and 24 h	↑ mRNA (only at 24 h) ↑ protein (all timepoints)	94
	Mouse	—	C2C12 myotubes	—	30 μM in medium	4, 8, 12 and 24 h	↔ mRNA or protein (all timepoints)	94
**Metformin+Vitamin D**	Rat	6 wks	Gastrocnemius	Two weeks HFD with 1 IP injection of STZ to induce T2D	Metformin: 100 mg/kg BW via oral gavage Vitamin D: 0.5 μg/kg BW via IP injection	56 d	↑ mRNA (T2D group)	110

Abbreviations: mRNA; Messenger RNA, BW; Body weight, LLC; Lewis Lung Carcinoma, HFD; High fat diet, IP; Intraperitoneal, STZ; Streptozotocin, T2D; Type 2 diabetes mellitus.
